# Fe(III) Is Essential for Porcine Embryonic Development via Mitochondrial Function Maintenance

**DOI:** 10.1371/journal.pone.0130791

**Published:** 2015-07-10

**Authors:** Ming-Hui Zhao, Shuang Liang, Seon-Hyang Kim, Xiang-Shun Cui, Nam-Hyung Kim

**Affiliations:** 1 Department of Animal Science, Chungbuk National University, Cheongju, Chungbuk, Republic of Korea; 2 Brain Korea 21 Center for Bio-Resource Development, Cheongju, Chungbuk, Republic of Korea; Institute of Zoology, Chinese Academy of Sciences, CHINA

## Abstract

Iron is an important trace element involved in several biological processes. The role of iron in porcine early embryonic development remains unknown. In the present study, we depleted iron (III, Fe^3+^) with deferoxamine (DFM), a specific Fe^3+^ chelator, in cultured porcine parthenotes and monitored embryonic development, apoptosis, mitochondrial membrane potential, and ATP production. Results showed biphasic function of Fe^3+^ in porcine embryo development. 0.5 μM DFM obviously increased blastocyst formation (57.49 ± 2.18% vs. control, 43.99 ± 1.72%, *P < 0*.*05*) via reduced (*P < 0*.*05*) production of reactive oxygen species (ROS), further increased mitochondrial membrane potential and ATP production in blastocysts (*P < 0*.*05*). 0.5 μM DFM decreased mRNA expression of *Caspase 3* (*Casp3*) and increased *Bcl-xL*. However, results showed a significant reduction in blastocyst formation in the presence of 5.0 μM DFM compared with the control group (DFM, 21.62 ± 3.92% vs. control, 43.99 ± 1.73%, *P < 0*.*05*). Fe^3+^ depletion reduced the total (DFM, 21.10 ± 8.78 vs. control, 44.09 ± 13.65, *P < 0*.*05*) and increased apoptotic cell number (DFM, 11.10 ± 5.24 vs. control, 2.64 ± 1.43, *P < 0*.*05*) in the blastocyst. An obvious reduction in mitochondrial membrane potential and ATP level after 5.0 μM DFM treatment was observed. Co-localization between mitochondria and cytochrome *c* was reduced after high concentration of DFM treatment. In conclusion, Fe^3+^ is essential for porcine embryonic development via mitochondrial function maintenance, but redundant Fe^3+^ impairs the function of mitochondria.

## Introduction

Iron plays an important role in cellular function in all organs and systems, particularly in rapidly growing and differentiating cells. In mammalian cells, iron is required for a variety of biochemical processes. It is a essential cofactor for non-heme enzymes for DNA synthesis and a vital component of the heme in hemoglobin, myoglobin and cytochromes [1]. Iron deficiency is known to cause multiple problems, including ventricular dilation [2], mitochondrial DNA damage [3], and hypertension in rat offspring [4]. In addition, nutritional iron deficiency is thought to trigger multiple cardiovascular diseases, including cardiac hypertrophy and chronic heart failure [5, 6].

Animals absorb iron from their diets, which contain two different forms of iron: inorganic non-heme iron in vegetables and grains and heme iron in red meat. Dietary non-heme iron exists mainly as iron (III, Fe^3+^), which binds to transferrin (Tf). The majority of cells absorb iron by Tf-mediated uptake via the transferrin receptor (TfR)-1. Iron uptake is roughly proportional to the number of TfRs on the cell surface. In erythroid cells, the low pH generated through the activity of a proton pump in endosome decreased the affinity of Tf for iron, and resulted in the release of Fe3^+^ from Tf in the endosome [7, 8]. Furthermore, Fe^3+^ is reduced to Fe^2+^ by a ferrireductase in the endosomal membrane, the six transmembrane epithelial antigen of the prostate 3 [9, 10].

Mitochondria are major powerhouses in all eukaryotic cells, producing ATP through oxidative phosphorylation and the citric acid cycle. Mitochondrial function is tightly controlled by cytoplasmic iron levels. Iron deficiency results in mitochondrial swelling, cytochrome *c* release [2], and mitochondrial DNA damage in rats [3]. In addition to their well-established role in providing the cell with ATP, mitochondria are the source of iron-sulfur clusters [11, 12] and heme-prosthetic groups [13] that are utilized by proteins throughout the cell in various critical processes. The mitochondria also have decreased respiratory control and gluconeogenesis after iron deficiency [14]. Mitochondria from iron-deficient rats exhibit partial uncoupling of the oxidative phosphorylation process. Iron deficiency also inhibits aconitase activity by damaging the Fe-S cluster and decreasing ATP production [15].

In oocytes and embryos, free Fe^3+^ can be reduced by increasing the levels of apo-Tf during embryonic development [16]. However, the levels of iron in oocytes and embryos [17, 18], follicular and uterine fluid [19] indicate that an essential role of iron for development. Although the function of iron in somatic cells is well established, its function in embryos development remains unknown.

DFM is an efficient chelating agent available for the treatment of iron overload [20, 21]. Solution in a sterile aqueous containing antiseptics was reportedly stable up to one week [22]. The toxicity of DFM on embryos did not be reported. However, intraperitoneal injection of DFM at 176 mg/kg (~250mmol/kg) to pregnant mice per day did not give development toxicity [23].

It is difficult to obtain pig embryos of homogeneous quality due to the relatively high incidence of polyspermy during in vitro fertilization [24]. Therefore, diploidparthenotes have frequently been used to study early development in the pig. In the present study, we evaluated the function of Fe^3+^ in porcine parthenotes development. To this end, we reduced the Fe^3+^ content in the embryos by DFM. The effect of DFM treatment was assayed by determining the embryonic development, ROS content, mitochondrial membrane potential, apoptosis and cytochrome *c* localization. To the best of our knowledge, this is the first report to address the function of Fe^3+^ in porcine embryos.

## Materials and Methods

Unless otherwise indicated, all chemicals were purchased from Sigma Chemical Company (Sigma—Aldrich, St. Louis, MO, USA).

### *In vitro* maturation (IVM) of porcine oocytes

Ovaries from prepubertal gilts were obtained from a local slaughterhouse (Farm story dodarm B&F, Umsung, Chungbuk, Korea) and transported to the laboratory at 37°C within 3 h of slaughter. Cumulus oocyte complexes (COCs) were aspirated from follicles ranging in diameters between 3 and 8 mm by a syringe with 18-gauge needle. Only COCs surrounded by a minimum of three layers of cumulus cells were selected for further studies. After aspiration, COCs were washed three times in TL-HEPES supplemented with 0.1% polyvinyl alcohol (PVA) and 0.05 g/L gentamycin. COCs were then cultured in TCM-199 medium supplemented with 0.1 g/L sodium pyruvate, 0.6 mM L-cysteine, 10 ng/mL epidermal growth factor, 10% porcine follicular fluid, 0.5 IU/mL luteinizing hormone, and 0.5 IU/mL follicle stimulating hormone for 44h. After IVM, oocytes were denuded by pipetting with 0.1% hyaluronidase. Denuded oocytes were collected for additional experiments.

### Parthenogenetic activation and embryo culture

After maturation, denuded oocytes were parthenogenetically activated by two 1.1kV/cm DC pulses for 50 μs followed by 3h incubation in PZM-5 medium [25, 26] containing 7.5 μg/mL cytochalasin B. Embryos were cultured in IVC medium (PZM-5 supplemented with 0.4% bovine serum albumin BSA, w/v and 0.6mM L-cysteine) under light mineral oil for 7 days at 38.5°C in 5% CO_2_ (v/v). To evaluate the effect of DFM on embryonic development, various concentration levels of DFM were added to the medium.

### Transferase-mediated dUTP nick-end labeling (TUNEL) assay

For evaluation of apoptosis in blastocyst, approximately 15 blastocysts from each group were washed three times in Dulbecco’s phosphate-buffered saline (dPBS) containing 0.1% PVA (dPBS/PVA) and then fixed in 3.7% paraformaldehyde (w/v) for 1 h at room temperature. After fixation, the blastocysts were permeabilized with 0.5% Triton X-100 (v/v) for 1 h at 38.5°C. Permeablized blastocysts were then incubated in fluorescein-conjugated deoxyuridine triphosphate (dUTP) and terminal deoxynucleotidyl transferase (Roche, Mannheim, Germany) in the dark for 1 h at 37°C. After nick labeling, the blastocysts were counterstained with 10 μg/mL Hoechst 33342 for 10 min at room temperature to label nuclei, followed by simple washing in dPBS/PVA, mounted under a coverslip, and examined under a fluorescence microscope (Nikon, Tokyo, Japan).

### Mitochondrial membrane potential assay

To measure mitochondrial membrane potential (Δφ_m_), blastocysts were washed three times with PBS and incubated in culture medium containing 0.5 μM 5,5′,6,6′-tetrachloro-1,1′,3,3′-tetraethyl-imidacarbocyanine iodide (JC-1) (Invitrogen, Grand Island, NY, USA) at 37°C in 5% CO_2_ for 30 min. Membrane potential was calculated as the ratio of red florescence, which corresponded to activated mitochondria (J-aggregates), to green fluorescence, which corresponded to less-activated mitochondria (J-monomers) [27]. Fluorescence was visualized with a Zeiss inverted confocal microscope equipped with a 40× oil immersion objective (Zeiss, Jena, Germany). Images were processed with ZEN software (Zen Software, Manchester, UK). The fluorescence intensity in the control group was arbitrarily set to 1, and the relative fluorescence intensity in the treatment groups was then measured. Three separate experiments were performed with 10–15 blastocysts in each.

### ATP content assay

The ATP content of 20 blastocysts was measured using a commercial assay (Invitrogen). Briefly, samples were washed three times with dPBS then transferred individually into 1 mL tubes on ice. The medium was removed, and blastocysts were lysed by freezing and thawing. Approximately 100 μl of ice-cold somatic cell reagent (FL-SAR) was added to each tube, and samples were incubated in an ice-water bath for 5 min. Thereafter, 100 μl of ice-cold assay buffer (diluted 1:25 with ATP assay buffer, FL-AAB) was added, and the tubes were maintained at room temperature for 5 min under limited light conditions. The ATP concentration was measured using a luminometer (Berthold, Wildbad, Germany) with a sensitivity of 0.01 pM. The ATP concentration in the control group was arbitrarily set to 1. Three separate experiments were performed with three replicates in each.

### Real-time reverse transcript-polymerase chain reaction (real time RT-PCR)

mRNA extraction and cDNA synthesis were performed as previously described [28]. Briefly, mRNA was extracted from 10 blastocysts using Dynabeads mRNA Direct Kit (Dynal Biotech ASA, Oslo, Norway) followed by routine cDNA synthesis by reverse transcription (RT) of RNA using an oligo(dT)_12–18_ primer and SuperScript Reverse Transcriptase (Invitrogen) following the manufacturer’s instructions.

Real-time RT-PCR was performed using the five primer pairs listed in Table 1. Real-time RT-PCR was performed in a Bio-Rad PCR machine (Bio-Rad, Hercules, CA, USA). Relative gene expression was analyzed using the 2-_ΔΔ_Ct method [29]. *Gapdh* mRNA was used as an internal control. Three independent experiments were performed with triplicate samples in each case.

**Table 1:** Primers used for real-time reverse transcription-PCR.

Gene	Primer sequences (5'-3')	Annealing temperature (°C)	Product size (bp)
*Bax*	F: CGGGACACGGAGGAGGTTT	60	189
	R: CGAGTCGTATCGTCGGTTG		
*Bcl2*	F: GAAACCCCTAGTGCCATCAA	60	196
	R: GGGACGTCAGGTCACTGAAT		
*Bcl-xL*	F: CTTACCTGAATGACCACCTAGAGC	60	182
	R: CCGACTGAAGAGCGAACCC		
*Casp3*	F: ACTGTGGGATTGAGACGG	55	110
	R: GGAATAGTAACGAGGTGCTG		
*Gapdh*	F: GCTTGCCTCCAGTGTCCTC	55/60	179
	R: GGCGTTGGCGATTTCAT		

F, forward; R, reverse.

### Real-time RT-PCR with Taqman for miRNA (miR) analysis

TaqMan microRNA assays were used to quantitate the miRNAs in the present study according to the conditions described previously [30]. Briefly, each 15 μl RT reaction contained purified 5 μl of RNA, 3 μl stem-loop RT primer, 1× RT buffer, 0.25 mM of each dNTP, 3.33 U/mL MultiScribe reverse transcriptase, and 0.25 U/mL RNase inhibitor. The reactions were incubated for 30 min at 16°C, 30 min at 42°C, 5 min at 85°C and then held at 4°C in an Applied Biosystems 6 thermocycler. Real-time RT-PCR for each microRNA assay was carried out in a 20 μL reaction that included 4 μl RT product, 1× TaqMan Universal PCR Master Mix, and 1 μL of 20× real-time solution containing TaqMan probe and primers. The amplification parameters for real-time RT-PCR were followed as set out in the manufacturer’s protocol. The threshold cycle (Ct) is defined as the fractional cycle number at which the fluorescence exceeds the fixed threshold of 0.2. U6 snoRNA was used as an internal control. Each experiment was repeated at least three times with three blastocysts per repeat. All primers, including those used for specific miRNA and cDNA synthesis and PCR amplification, and the kit used for miRNA analyses were purchased from Applied Biosystems (Bedford, MA, USA).

### ROS content assay

In the experiments, ROS content of the treated and untreated embryos was measured at the blastocyst stage. The ROS content was quantified using the dichlorodihydrofluorescein diacetate (DCHFDA, Molecular Probes, Invitrogen) method, as previously described [31]. Live imaging and quantitation were conducted on a fluorescence microscope (Nikon, Tokyo, Japan) by using photoshop (CS2, Adobe, USA).

### Evaluation of co-localization of mitochondrial and cytochrome *c*

Blastocysts were stained for mitochondria according to the method descried in the previous study [32]. Briefly, blastocysts were incubated in IVC medium supplemented with 0.5 μmol/L MitoTracker Red CMXRos (Molecular Probes, Eugene, OR) for 30 min in incubator with 5% CO_2_ for 30 minutes at 38.5°C, followed by three washes with dPBS/PVA for 20 minutes. Then the blastocysts were fixed in 3.7% (w/v) paraformaldehyde in dPBS/PVA at room temperature. After 1h of permeabilization in PBS containing 0.1% Triton X-100 at 38.5°C, blastocysts were then blocked with 3% BSA in dPBS for 1h. They were then labeled with 100 μg/ml anti-cytochrome *c* antibody (Abcam) in blocking solution overnight in 4°C then labeled by rhodamine labeled second antibody. Nuclear was staining with 10 μg/ml Hoechst 33342 in PBS for 10 minutes, followed by washing three times in PBS, and then mounted on glass slides. Blastocyst were observed with a laser-scanning confocal microscope (Zeiss LSM 710 META, Germany). Co-localization of mitochondrial and cytochrome *c* were evaluated by Person’s Correlation coefficient.

### Statistical analysis

All data were analyzed using SPSS software version 11.0 (SPSS Inc., Chicago, IL USA). JC-1, DCDHF signal intensity, and gene expression were analyzed by one-way ANOVA. The percentages of embryos that developed to a particular stage were determined by Chi-square tests. *P < 0*.*05* was considered statistically significant.

## Results

### Effect of different DFM concentrations on porcine parthenotes development

**Fig 1:**
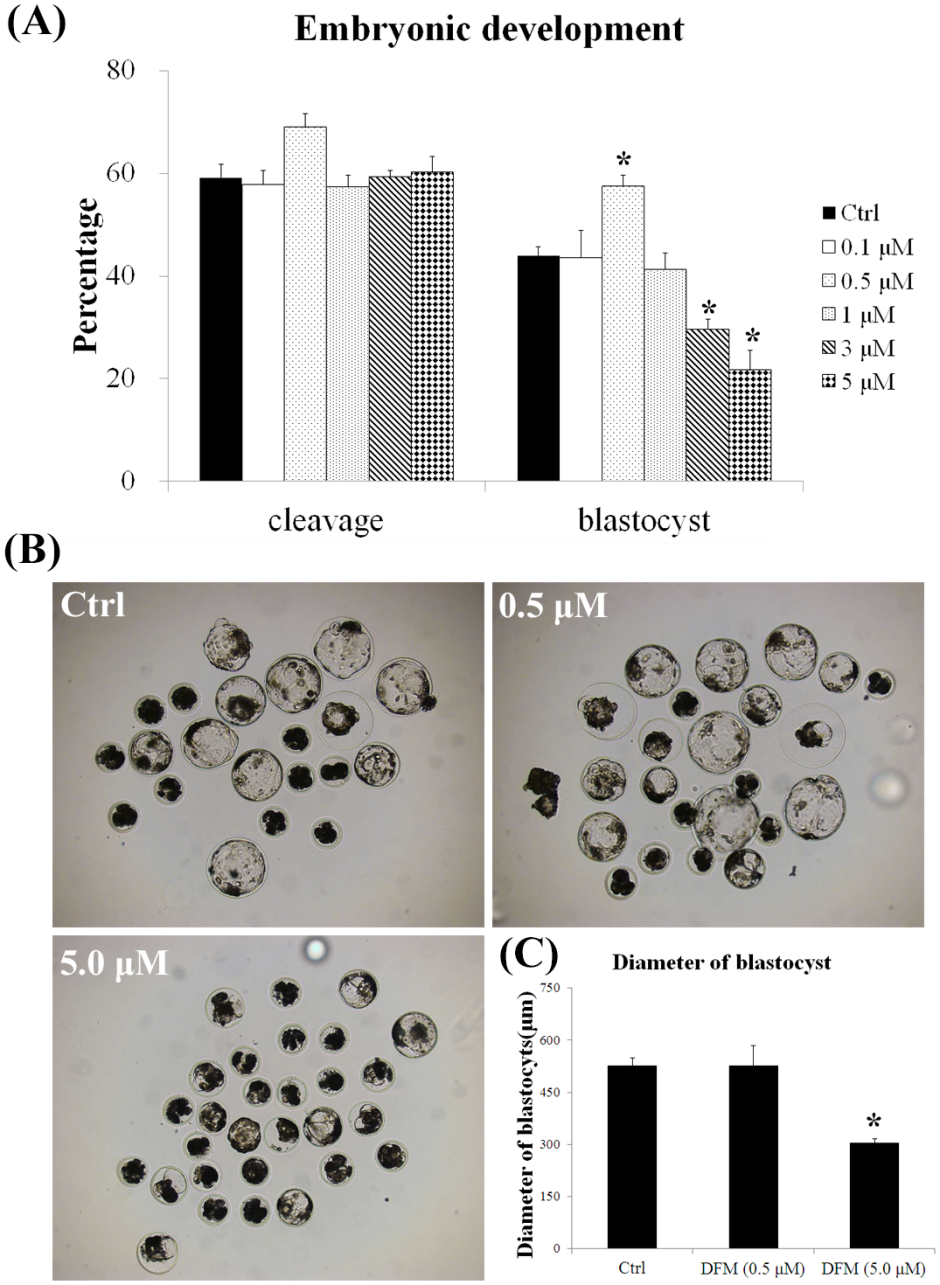
Cleavage and blastocyst formation after DFM treatment. (A), Cleavage and blastocyst formation of porcine parthenotes cultured in the presence of different concentration of DFM. (B), Morphology and diameter of blastocysts cultured in the absence or present of 0.5 and 5.0 μM DFM. (C), The diameters were measured by image pro-plus software. Values represent means ± SEM from at least three separate experiments. Scale bar = 500 μm, *P < 0.*05*.

To determine the effect of DFM on embryo development, after maturation, oocytes were artificially activated and cultured in the presence of 0, 0.1, 0.5, 1.0, 3.0, and 5.0 μM DFM for 7 days. The results were shown in Fig 1. No obvious difference in cleavage rate was observed. Interestingly, 0.5 μM of DFM (57.49 ± 2.18%, n = 130) significantly enhanced blastocyst formation after *in vitro* culture compared with the 0.1 μM (43.64 ± 5.19%, n = 131) group and control group (43.99 ± 1.72%, n = 167, *P < 0*.*05*). However, 3.0 μM (29.58 ± 1.97%, n = 132) and 5.0 μM (21.62 ± 3.91%, n = 106) of DFM sharply reduced blastocyst formation (*P < 0*.*05*).After 5.0 μM of DFM treatment, the diameter of blastocyst was decreased in the treatment group (304.78 ± 12.17 μm, n = 29, *P < 0*.*05*) compared to control (526.15 ± 23.67 μm, n = 26). However, there was no significant difference between 0.5 μM of DFM and control groups (Fig 1B and 1C).

### Effect of Fe^3+^ depletion on total cell number and apoptosis in blastocysts

**Fig 2:**
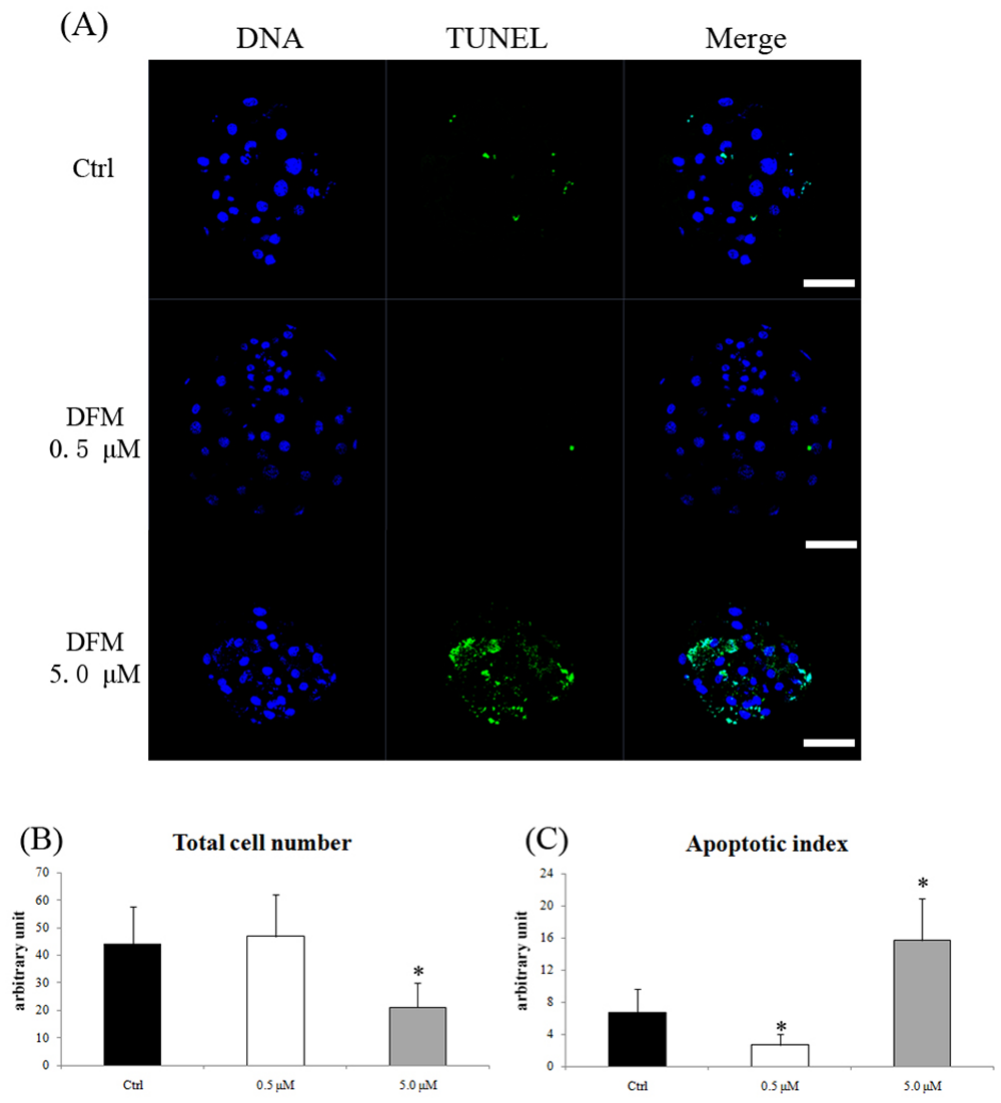
Total cell number and apoptosis after DFM treatment. (A), Total cell number was determined by Hoechst 33342 (blue) staining. Apoptosis was determined by TUNEL (green) staining. (B) and (C), The number of total and apoptotic cells in porcine blastocysts developed *in vitro*. Black bar, control group; white bar, DFM 0.5 μM; gray bar, DFM 5.0 μM. Values represent means ± SEM from at least five separate experiments. *P < 0.*05*.

To evaluate blastocyst quality, the total cell number and incidence of apoptosis were counted. 0.5 μM of DFM did not affect the total cell number of blastocysts. However, 5.0 μM DFM treatment significantly decreased the total cell number in blastocysts (21.10 ± 8.78, n = 90 vs. control, 44.09 ± 13.65, n = 91, *P < 0*.*05*). TUNEL staining showed that the number of apoptotic cells was significantly lower in 0.5 μM but higher in the 5.0 μM of DFM group than in the control (*P < 0*.*05*, Fig 2).

### Effect of Fe^3+^ depletion on expression of apoptosis-related genes

**Fig 3:**
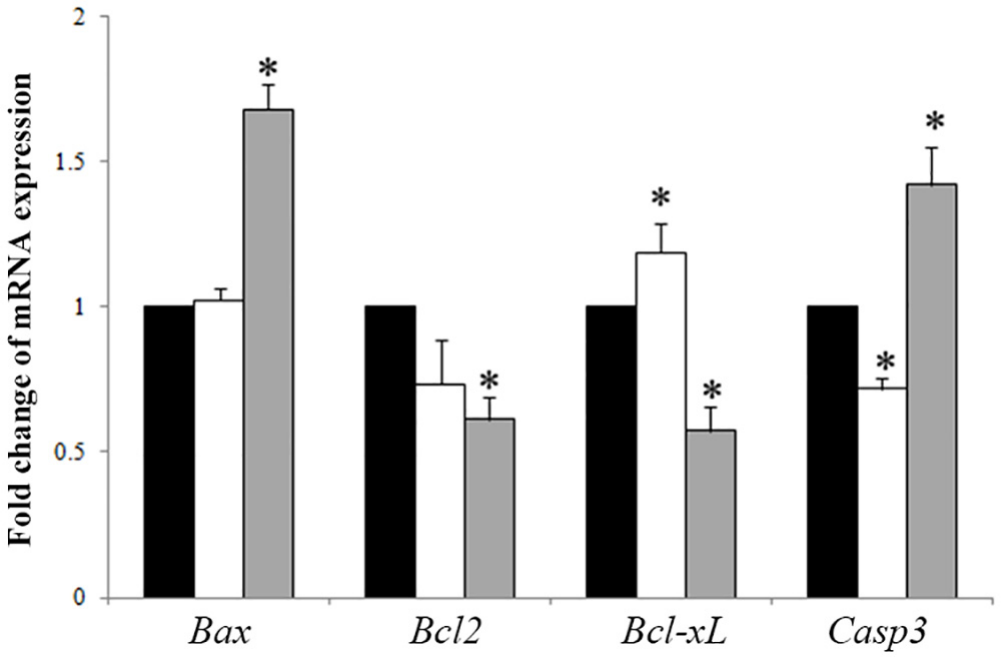
Expression of apoptosis related genes in porcine blastocysts cultured for seven days. mRNA was extracted from blastocysts cultured in the absence or presence of DFM. Expressions of anti-apoptotic and apoptosis-related genes were analyzed by real-time RT-PCR. Black bar, control group; white bar, DFM 0.5 μM; gray bar, DFM 5.0 μM. *P < 0.*05*. Values are the mean ± SEM of 3–4 independent experiments.

To clarify the molecular mechanism of apoptosis induction, mRNAs of apoptosis-related genes were evaluated by real-time RT-PCR. Results showed that low concentration (0.5 μM) of DFM significantly (*P < 0*.*05*) decreased the expression of apoptosis-related gene *Casp3*. Compared with the control group, the expression of the anti-apoptosis gene *Bcl-xL* was significantly increased in blastocysts. However, there were no significant differences in the expression of *Bax* and *Bcl 2* (Fig 3).

We compared the expression of apoptotic related genes in the blastocysts after high concentration of DFM (5.0 μM) treatment. Fe^3+^ depletion significantly increased the mRNA expression of apoptotic genes *Bax* and *Casp3* (*P < 0*.*05*), while the expression of the anti-apoptotic genes *Bcl2* and *Bcl-xL* were decreased (*P < 0*.*05*, Fig 3).

**Fig 4:**
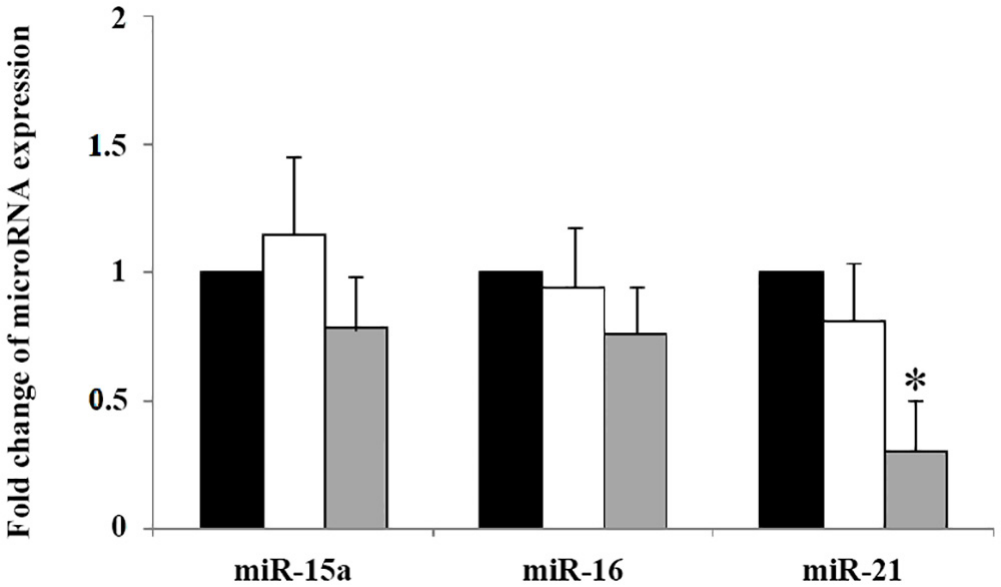
Expression of apoptosis related miRNAs in porcine blastocysts cultured for seven days. Apoptosis-related miRNAs were analyzed by TaqMan real-time RT-PCR. Black bar, control group; white bar, DFM 0.5 μM; gray bar, DFM 5.0 μM. *P < 0.*05*. Values are the mean±SEM of 3–4 independent experiments.

Fe^3+^ depletion decreased the expression of miR-21 after 5.0 μM of DFM treatment (*P < 0*.*05*), but there were no significant differences in miR-15a and miR-16 expression levels in the all groups (Fig 4).

### Effect of Fe^3+^ depletion on mitochondrial membrane potential and ATP production

**Fig 5:**
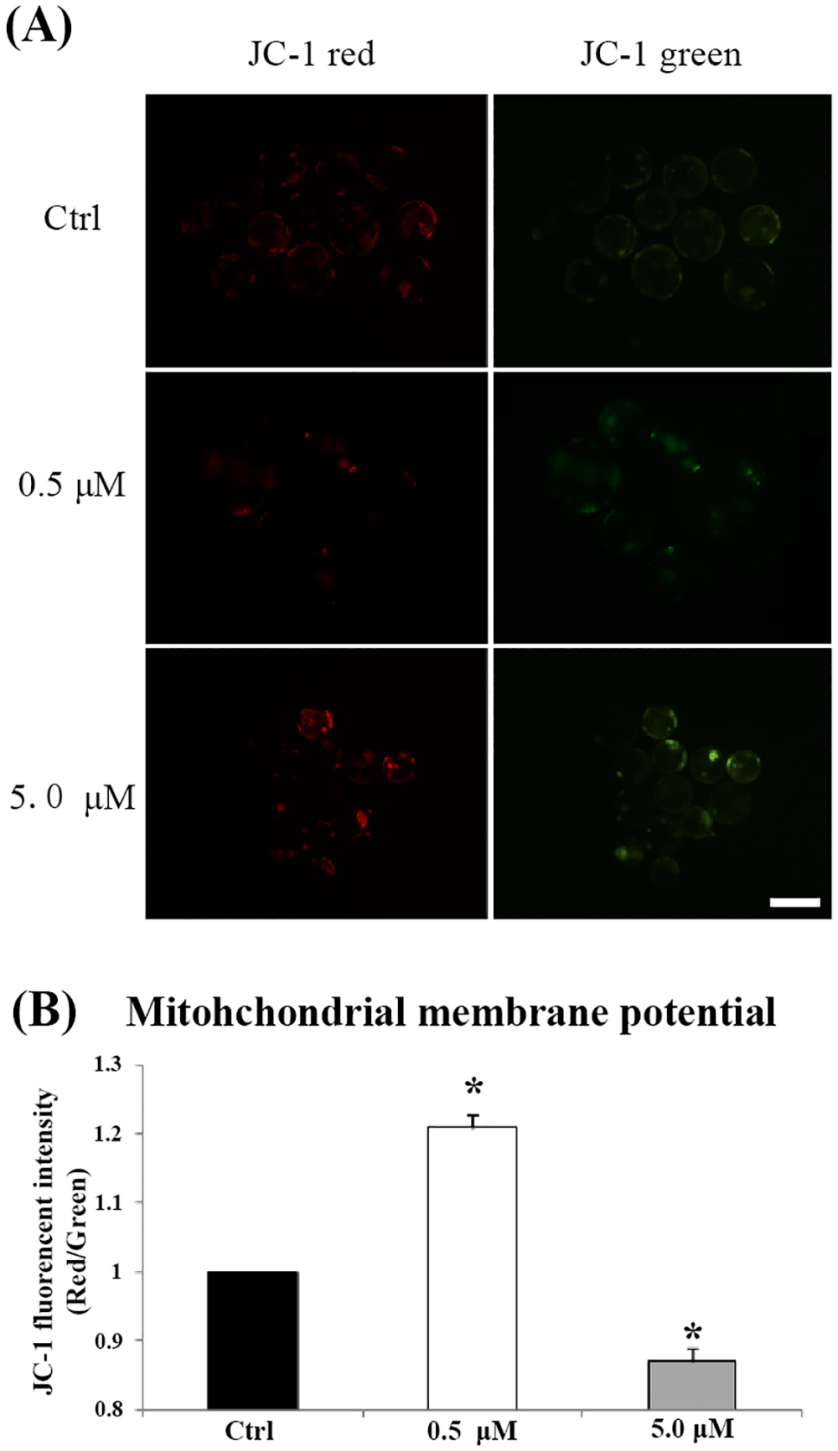
Membrane potential in blastocyst cultured in the absence or presence of DFM. (A), Membrane potential was calculated as the ratio of red florescence, which corresponds to activated mitochondria (J-aggregates), to green fluorescence, which corresponds to less-activated mitochondria (J-monomers). (B), Fluoresces of each blastocyst were analyses by image pro plus software. The control was arbitrarily set at 1. Black bar, control group; white bar, DFM 0.5 μM; gray bar, DFM 5.0 μM. Values represent means ± SEM from at least three separate experiments. Scale bar = 500 μm. *P < 0.*05*.

**Fig 6:**
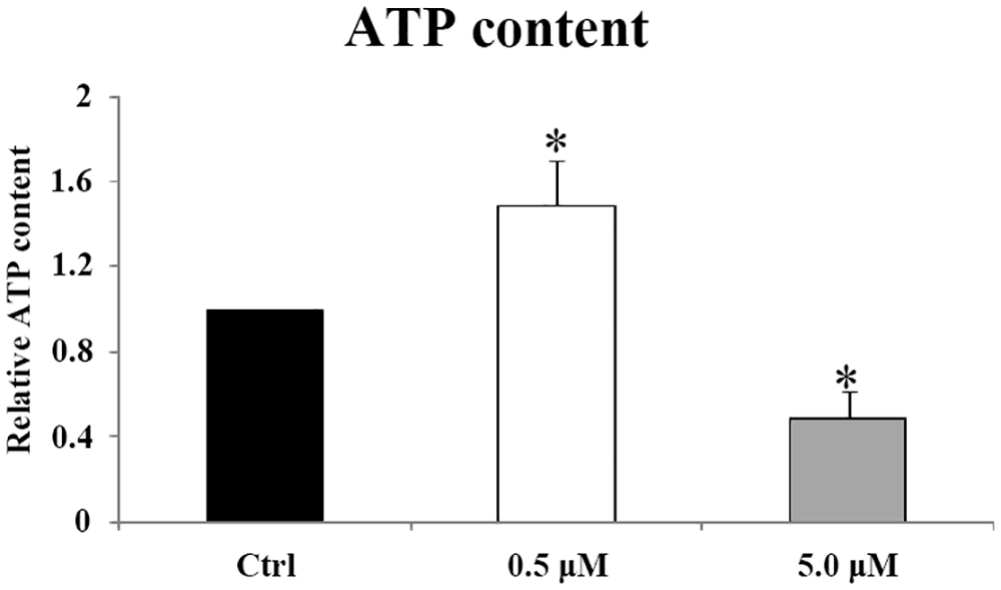
ATP content in blastocyst cultured in the absence or presence of DFM. The control was arbitrarily set at 1. Black bar, control group; white bar, DFM 0.5 μM; gray bar, DFM 5.0 μM. Values represent means ± SEM from at least three separate experiments. *P < 0.*05*.

To explore the mechanism by which Fe^3+^ depletion affected development of porcine parthenotes, the mitochondrial membrane potential and ATP content in blastocysts were checked. The results were shown in Figs 5 and 6. After low concentration (0.5 μM) of DFM treatment, the mitochondrial membrane potential was significantly increased (*P < 0*.*05*) compared to the control group. Similarly, the ATP content in DFM treated blastocysts was higher (*P < 0*.*05*) than in the control group. However, high concentration (5.0 μM) of DFM treatment not only reduced mitochondrial membrane potential, but also decreased the ATP content in blastocysts (*P < 0*.*05*).

### Effect of DFM on ROS content in the porcine blastocysts

**Fig 7:**
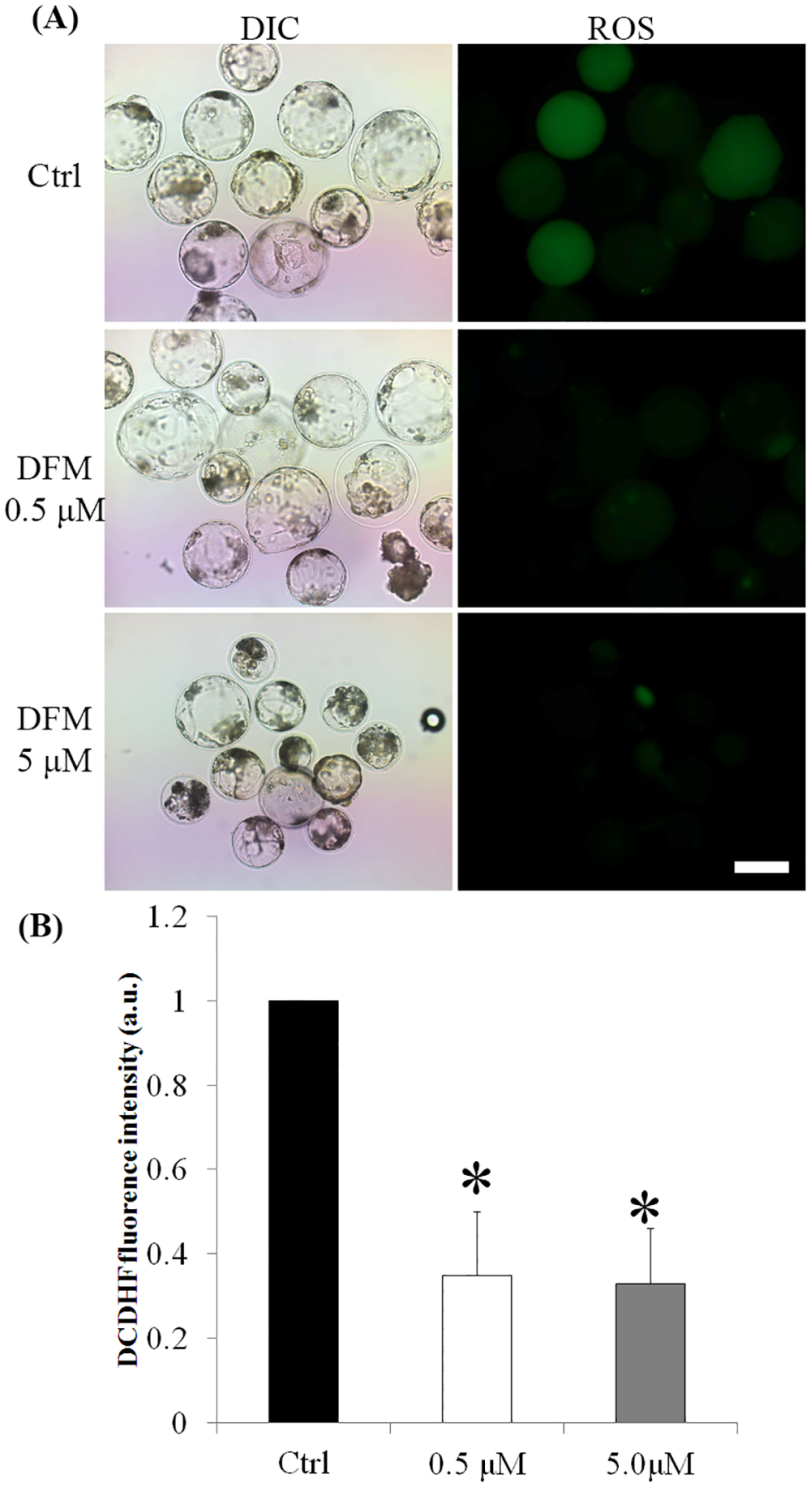
ROS content in blastocysts cultured in the absence or presence of DFM. (A), ROS in blastocysts were stained by DCDHF (green). (B), Fluorescence intensity was analyzed by Photoshop. Data corresponding to the control was arbitrarily set at 1. Black bar, control group; white bar, DFM 0.5 μM treatment group; gray bar, DFM 5.0μM treatment group. Values represent mean ± SEM from at least three separate experiments. Scale bar = 500 μm *, *P < 0*.*05*.

To assess why DFM enhanced mitochondrial membrane potential, ROS content in blastocyst was determined. The results showed that relatively lower ROS content was found in the blastocysts which developed in the presence of 0.5 and 5 μM DFM. Higher DCDHF signal was observed in the blastocysts cultured in the absence of DFM. Fluorescence intensity in the DFM group was significantly lower (*P < 0*.*05*) than that in the control. However, there was no obvious difference of ROS content between 0.5 and 5 μM DFM groups (Fig 7).

### Effect of DFM on cytochrome *c* release

**Fig 8:**
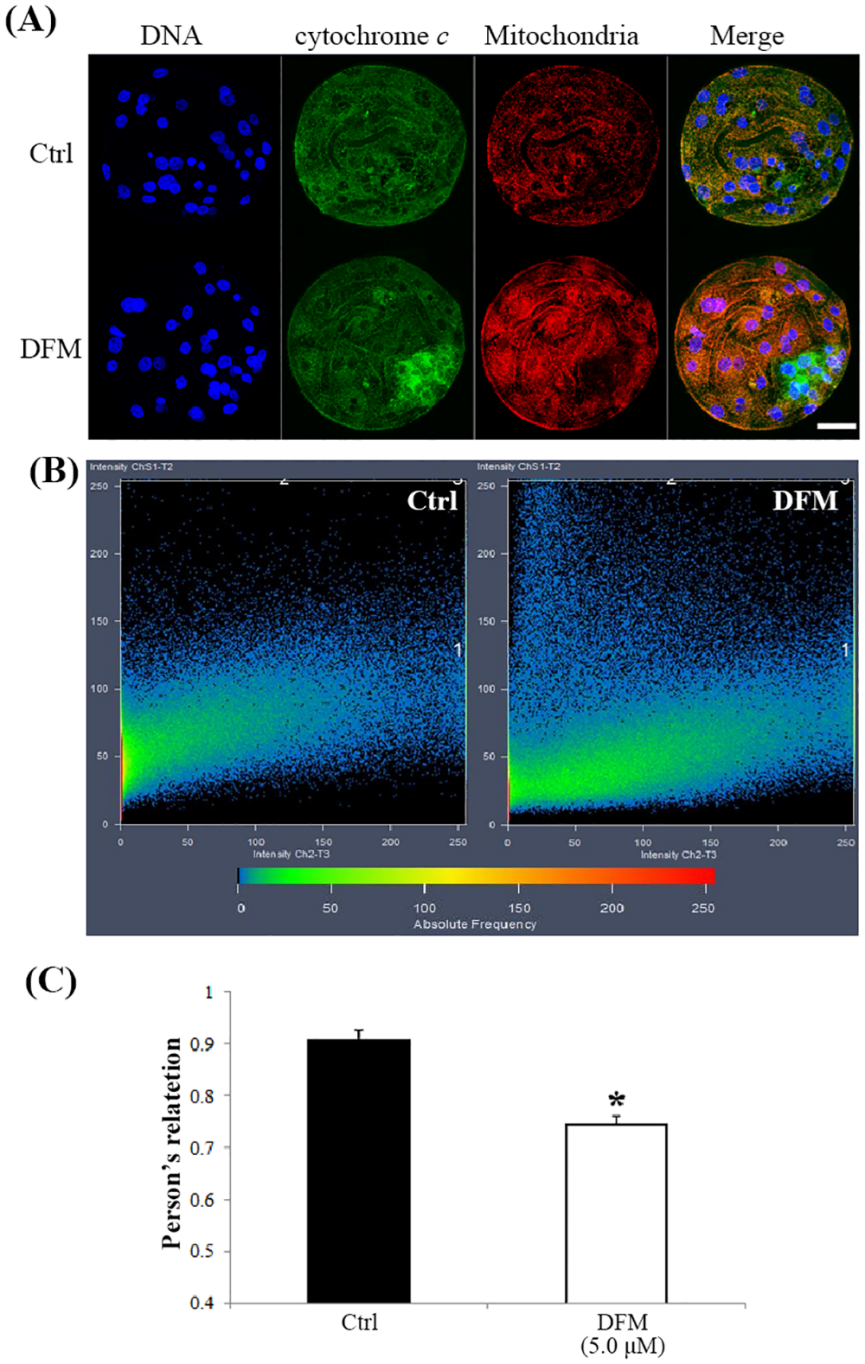
Co-localization of cytochrome *c* and mitochondria in blastocysts cultured for seven days in the absence or presence of DFM. (A), Blastocysts were labeled with a cytochrome *c*-specific antibody (green) and MitoTracker (red). (B), Co-localization was analyzed using Image pro plus. (C), Pearson’s R was used to compare the co-localization of cytochrome *c* and mitochondria. Black bar, control group; white bar, DFM 5.0 μM. Scale bar = 200μm, *P < 0.*05*. Values are the mean ± SEM of 3–4 independent experiments.

To explore the apoptotic pathway, the co-localization of mitochondria and cytochrome *c* was determined in control and high DFM treated blasotysts. Pearson’s R was employed to evaluate co-localization. Pearson’s R in Fe^3+^-depleted blastocysts was significantly reduced, indicating a poor co-localization of mitochondrial and cytochrome *c* than that in the control group, which displayed stronger co-localization of cytochrome *c* and MitoTracker (Fig 8).

## Discussion

**Fig 9:**
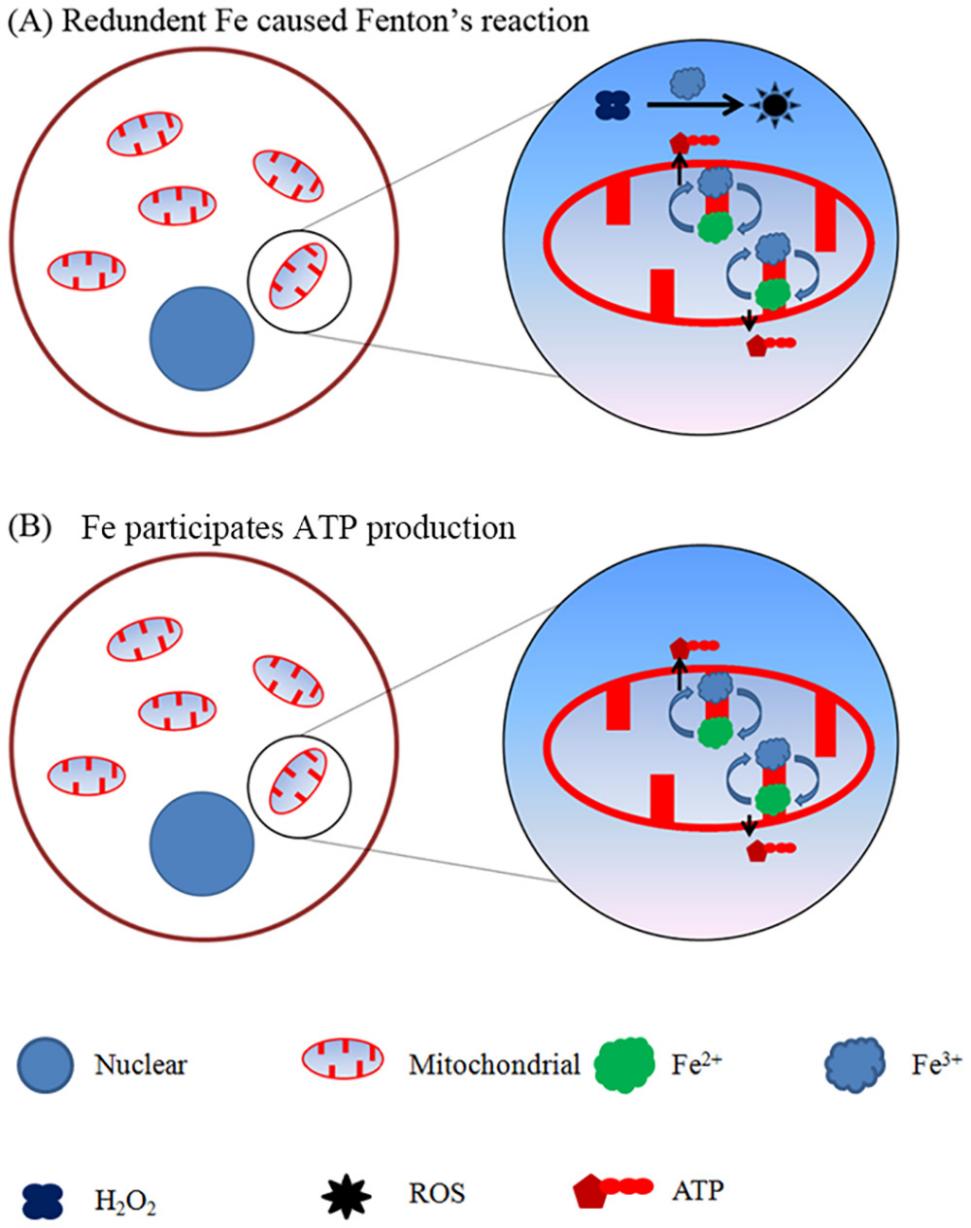
A working model for roles of Fe^3+^ in the porcine parthenotes development. (A), Redundant Fe^3+^ caused higher concentration of ROS in cytoplasm, thus depletion of redundant Fe^3+^ reduced ROS in the porcine parthenotes. (B), Fe^3+^ is essential for ATP production.

In the present study, we found that Fe^3+^ plays biphasic roles in porcine parthenotes development (Fig 9). Our results shown that redundant iron resulted in high concentration of ROS, depletion of that redundant iron significantly reduced ROS content, and further protected function of mitochondria (Fig 9A). However, over-depletion of Fe^3+^ impaired the function of mitochondria, thus resulted in apoptosis in the blastocysts (Fig 9B).

Although reduced free cytoplasmic iron increased embryo formation [16] by preventing production of ROS through Fenton’s reaction [33], iron participates in several biological processes. To determine the function of iron, we depleted free Fe^3+^ with high concentration of DFM. After depletion, blastocyst development was reduced in a dose-dependent manner and diameters were decreased in iron-deficient blastocysts. Mammalian blastocyst formation is dependent on establishment of trophectoderm (TE) ion and fluid transport mechanisms [34]. The Na^+^/K^+^ ATPase is also a critical mediator of blastocyst formation as it establishes a transtrophectoderm ionic gradient that directs fluid movement across the TE epithelium [35, 36], which directly results in blastocyst cavity formation. Na^+^/K^+^ ATPase function is closely related to ATP, which is produced by mitochondria. Based on this information, we hypothesized that iron deficiency in embryos affects the function of mitochondria.

To confirm the mechanism of low blastocyst formation and increased apoptosis, t ATP contents of blastocysts were determined. ATP production is the most important function of mitochondria. In the present experiment, the results showed that iron deficiency reduced ATP level in blastocysts. This finding might explain the reduction in blastocyst formation and diameter. Reduced ATP content after iron depletion also reflects the dysfunction of mitochondria.

To clarify the reason for lower ATP production after iron deficiency, we investigated mitochondrial function by measuring the mitochondrial membrane potential, which is critical for the production of ATP. During oocyte maturation, there is a significant increase in mitochondrial membrane potential [37]. Our previous study showed that decreased blastocyst formation is always accompanied by decreased mitochondrial membrane potential [38, 39], which agrees with the results presented herein.

The mechanism by which iron depletion reduces membrane potential is still unknown. However, ATP production is dependent on electron transfer in the mitochondria, which involves the Fe^2+^ and Fe^3+^ transition. In the electron transport chain, many Fe-S proteins participate in electron transfer. Formation of mitochondrial membrane potential is mediated by the proton gradient established by four complexes located on the mitochondrial membrane, indicated that iron depletion probably impairs the function of the four complexes, further blocking electron transfer and resulting in decreased membrane potential and ATP content.

Iron deficiency leads to serious apoptosis in blastocysts, which can be explained by the dysfunction of mitochondria. Mitochondria play a key role in the apoptotic process [40, 41]. Mitochondrial control of apoptosis has been described at several levels, including ATP production [42], mitochondrial membrane potential, and mitochondrial membrane permeability for the release of apoptogenic factors from the intermembrane space into the cytosol [43]. In the present experiment, co-localization analysis of cytochrome *c* and mitochondria showed that Fe^3+^ depletion induced release of cytochrome *c* from mitochondria. In mammalian cells, cytochrome *c* initiates a major *Caspase* activation pathway. In this pathway, a variety of apoptotic stimuli cause cytochrome *c* release from the mitochondria, which in turn induces a series of biochemical reactions that result in C*aspase* activation and subsequent cell death [44]. The released cytochrome *c* may explain the increased expression of *Bax* It belongs to the prodeath *Bcl*2 family proteins, which form the last gateway for cytochrome *c* release [45]. Released cytochrome *c* activates *Casp3* expression, which was increased in the present study.

Reduced mitochondrial metabolic capacity results in decreased the percentage of blastocyst formation following parthenogenetic activation in the present study. Although there are epigenetic differences between parthenote and IVF embryos, they share same basic metabolism mechanism.

In conclusion, the present study showed that iron is essential for embryonic development, which confirmed the function of mitochondria, however, redundant iron impaired function of mitochondria via induction of high concentration of ROS.

## Supporting Information

S1 Data Original data include cleavage rate, blastocyst rate, total cell number, apoptosis cell number, mitochondrial membrane potential, ATP content, cytochrome c localization and expressions for gene and microRNA. (XLSX)
